# *Lactobacillus rhamnosus* GG Administration Is Associated with Stimulation of Vitamin D/VDR Pathway and Mucosal Microbiota Modulation in Ulcerative Colitis Patients: A Pilot Study

**DOI:** 10.3390/ph18111651

**Published:** 2025-11-01

**Authors:** Cristiano Pagnini, Manuele Gori, Maria Carla Di Paolo, Riccardo Urgesi, Claudia Cicione, Maria Zingariello, Francesca Arciprete, Viola Velardi, Elisa Viciani, Antonella Padella, Andrea Castagnetti, Maria Giovanna Graziani, Gianfranco Delle Fave

**Affiliations:** 1 Gastroenterology and Digestive Endoscopy Department, S. Giovanni Addolorata Hospital, 00184 Rome, Italy; 2 Institute of Biochemistry and Cell Biology (IBBC)—National Research Council (CNR), International Campus “A. Buzzati-Traverso”, Via E. Ramarini 32, Monterotondo Scalo, 00015 Rome, Italy; 3 Laboratory of Regenerative Orthopaedic, Research Unit of Orthopaedic Surgery, Campus Bio-Medico University of Rome, Via A. del Portillo 21, 00128 Rome, Italy; 4 Unit of Microscopic and Ultrastructural Anatomy, Department of Medicine, Campus Bio-Medico University of Rome, Via A. del Portillo 21, 00128 Rome, Italy; 5 Wellmicro Srl, 40138 Bologna, Italy; 6 Department of Gastroenterology, “Sapienza” University of Rome, 00185 Rome, Italy; 7 S. Andrea ETS, 00199 Rome, Italy

**Keywords:** probiotic, *Lactobacillus rhamnosus* GG, LGG, vitamin D, vitamin D receptor, VDR, microbiota

## Abstract

**Background**: The interaction between probiotics and the vitamin D/vitamin D receptor (VDR) pathway has been increasingly explored as a potential mechanism for immune modulation in inflammatory bowel disease (IBD). *Lactobacillus rhamnosus* GG (LGG) has shown promising results in ulcerative colitis (UC) patients, but its effect on the VDR pathway remains unexplored in humans. **Aim**: To test the hypothesis that LGG can stimulate the vitamin D/VDR pathway and modulate mucosal-adherent microbiota. **Methods**: In this study, we analyzed a subgroup of 13 patients from the LGGinUC trial, in which UC patients with mild-to-moderate disease activity received LGG monotherapy for four weeks. Colonic biopsy samples were collected before and after treatment to evaluate VDR expression via RT-qPCR and immunohistochemistry. Mucosal-adherent microbiota was also analyzed by DNA extraction and next-generation sequencing (NGS). **Results**: LGG administration significantly increased VDR mRNA expression in colonic mucosa (*p* < 0.05), with a corresponding rise in VDR protein levels in both epithelial and sub-epithelial compartments. Microbiota analysis revealed a reduction in α-diversity, primarily due to a decrease in commensal bacterial species, while β-diversity remained largely unchanged. **Conclusions**: Although the present results have to be considered preliminary, this is the first human study demonstrating that probiotic supplementation can upregulate VDR expression in colonic mucosa. We propose that LGG may exert its beneficial effects in UC by stimulating the VDR pathway, which in turn modulates mucosal immunity and microbiota composition. Further studies with larger sample sizes and longer treatment durations are needed to validate these findings and explore their therapeutic implications.

## 1. Introduction

In recent decades, the so-called “microbiota revolution” has highlighted the crucial role of gut microbiota in various pathological conditions, including inflammatory bowel disease (IBD) [1]. These are chronic conditions, with unknown etiology and immunomediated pathogenesis, whose two main entities are represented by Crohn’s disease and ulcerative colitis (UC). In particular, the latter is a pathology characterized by exclusively colonic mucosal inflammation not overcoming the lamina propria layer, typically starting from the rectum and with a variable proximal extension, clinically presenting with intermitting phases of remission and flares [2]. The treatment aims to induce and maintain clinical and endoscopic remission, and comprises mesalamine, corticosteroids, immunosuppressants, biological drugs, and small molecules [3]. Despite many novel drugs having been approved and some more in the pipeline for market proposal, there are still unmet needs for a consistent proportion of patients not responding to therapy or experiencing adverse events. So there is still an urgent need for therapeutic options with a safe profile and proved efficacy. In this regard, the manipulation of gut microbiota through probiotic administration has been widely studied, and several bacterial strains have shown promising effects in both the induction and maintenance of remission in UC patients [4,5]. Among these, *Lactobacillus rhamnosus* GG (LGG) has been extensively investigated due to its remarkable adhesion properties, immunomodulatory potential, and ability to reinforce the intestinal barrier, making it a promising therapeutic option for UC patients [6]. Clinical studies have demonstrated that LGG is as effective as mesalamine in maintaining remission for up to one year and can induce remission as a monotherapy in mild-to-moderate UC patients [7,8]. Additionally, preclinical models have confirmed its anti-inflammatory effects, including the reduction in mucosal pro-inflammatory mediators such as TNFα and IL-17, enhancement of barrier integrity, and modulation of local immune responses [9,10,11,12]. Despite these promising findings, the precise mechanisms underlying the beneficial effects of probiotics remain incompletely understood, likely involving multiple pathways.

In parallel, vitamin D has emerged as a fundamental regulator of immune homeostasis beyond its well-established role in bone metabolism. The vitamin D receptor (VDR) is widely expressed in various cell types, particularly in the intestinal mucosa, where it plays a key role in both innate (epithelial cells, macrophages, dendritic cells) and adaptive (B and T lymphocytes, natural killer cells) immunity. The interaction between vitamin D and VDR contributes to maintaining mucosal immune balance by strengthening the intestinal barrier, modulating the composition of the gut microbiota, preventing dysbiosis, controlling local infections, and reducing systemic inflammation [13]. Alterations in this pathway have been implicated in the pathogenesis of several immune-mediated diseases, including IBD [14].

Recent studies have explored potential interactions between probiotics and the vitamin D pathway, suggesting a synergistic effect between probiotic administration and vitamin D supplementation in various pathological conditions [15]. However, the molecular mechanisms underlying this potential interaction remain unclear, and no human studies have demonstrated the direct effect of probiotics on VDR expression in vivo.

The aim of this study was to investigate the novel hypothesis that LGG exerts its beneficial effects in UC patients through the stimulation of the vitamin D/VDR pathway. Additionally, we sought to assess the impact of LGG on mucosal-adherent microbiota composition and diversity.

## 2. Results

### 2.1. Clinical Outcome

The LGGinUC trial included 76 UC patients with mild-to-moderate clinical activity who received LGG monotherapy at two different doses for four weeks, following a two-week washout period from mesalamine. LGG therapy was well tolerated and a consistent proportion of patients had amelioration of clinical symptoms (42% at the intention-to-treat analysis), with no significant difference between patients receiving different doses [8]. Among these, a subset of 13 patients (17%) underwent molecular investigations and were included in the present study. Colonic biopsy samples were collected both before and after LGG administration, and total DNA and RNA were extracted for analysis. No significant differences were observed between the total study population and the investigated subgroup in terms of demographic characteristics, disease phenotype, or clinical response to LGG therapy. In particular, 46% of patients exhibited a clinical response, defined as a reduction in the Partial Mayo score, and the majority (69%) showed stable endoscopic disease activity at the end of the study period (Table 1).

### 2.2. VDR Expression

**Figure 1:**
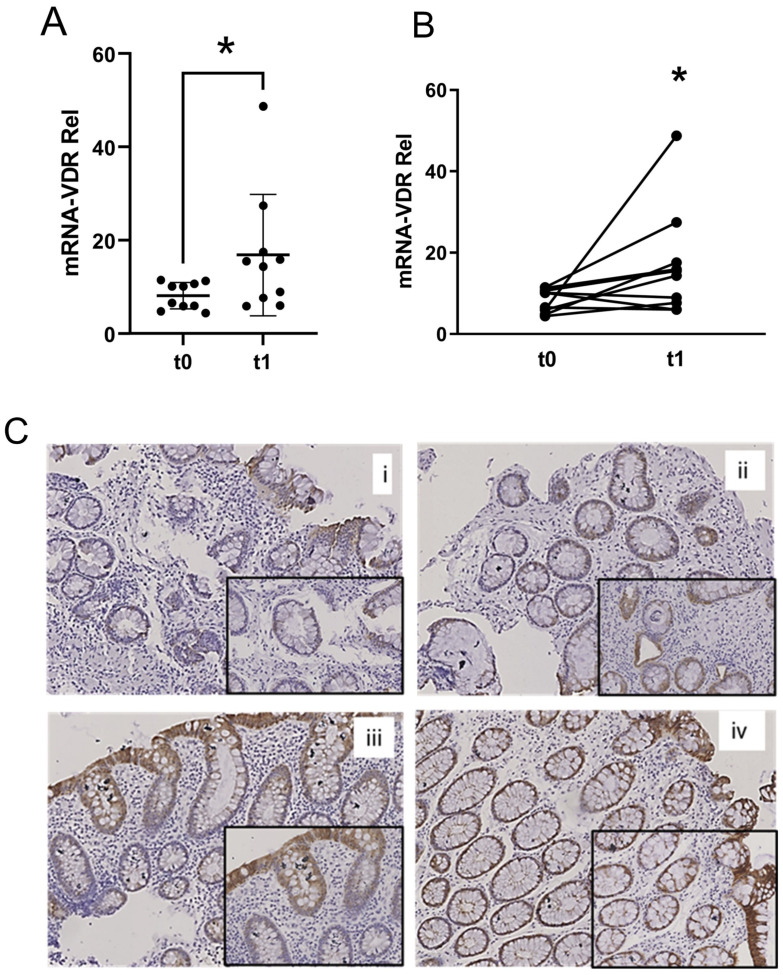
Increased expression and production of VDR after LGG administration. (**A**) VDR mRNA levels quantification before (t0) and after (t1) LGG treatment in total samples (*n* = 10 per group), difference was significant (*p* < 0.05) at Mann–Whitney U test; (**B**) VDR mRNA relative increment in the single individuals (*n* = 10 per group), difference was significant (*p* < 0.05) at Wilcoxon rank-sum test for paired data; and (**C**) VDR detection by immunohistochemical staining in histological sections (10× and 20× magnification), with two representative subjects before (**i**,**iii**) and after (**ii**,**iv**) LGG treatment represented. * *p* < 0.05.

Molecular analyses revealed that LGG administration significantly upregulated VDR expression in the colonic mucosa. VDR mRNA levels, quantified by real-time PCR (RT-qPCR) following total RNA extraction and cDNA transcription, were significantly increased after LGG treatment compared to baseline (8.1 ± 2.8 vs. 16.8 ± 13, *p* < 0.05, Figure 1A). When analyzed at the individual level, a significant relative increase in VDR expression was observed in all patients following LGG supplementation (mean increment 2.4 ± 2.2, *p* < 0.05, Figure 1B). Immunohistochemical staining confirmed a consistent increase in VDR protein production, particularly within the epithelial layer and sub-epithelial compartment (Figure 1C).

### 2.3. Mucosal Microbiota Composition

**Figure 2:**
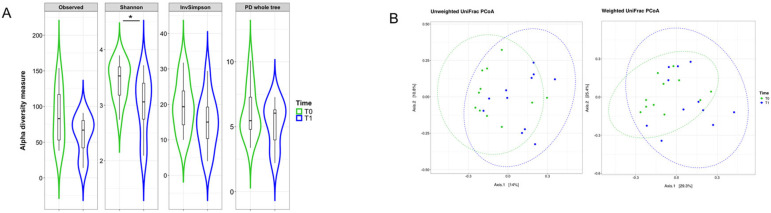
Mucosal microbiota evaluation after LGG treatment. (**A**) Violin plots showing the comparison of alpha diversity measures between T0 (green) and the end of the probiotic treatment T1 (blue). Observed = Observed Species; PD whole tree = Phylogenetic Diversity Whole Tree; Shannon = Shannon–Wiener index; InvSimpson = Inverse Simpson’s index. Median, first, third quartile and outliers are shown. We observed a significant reduction at Shannon Index (PERMANOVA test) after LGG administration. * *p* < 0.05. (**B**) Principal Coordinate Analysis (PCoA) on unweighted and weighted UniFrac distance metric calculated at T0 (green dots) and T1 (blue dots). Each sample is represented by a dot. Axis 1 explained 14% and 29.3% of the variation observed, while Axis 2 explained 10.8% and 25.4% of the variation, in the left and right graphs, respectively; dashed green ellipses, for T0 data, or blue ellipses, for T1 data, were calculated on the cluster of the sample data using the function ‘stat_ellipse’ and assuming a multivariate t–distribution; PERMANOVA on weighted and unweighted UniFrac Pr (>F) = n.s. (not significant).

**Figure 3:**
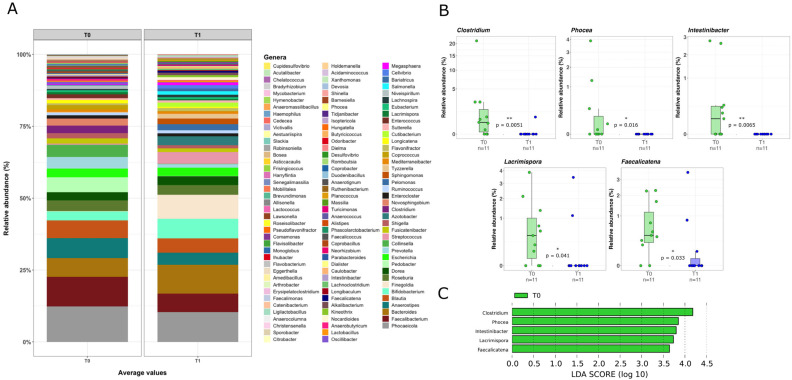
Qualitative analysis at genus level of the mucosal microbiota before and after LGG treatment. (**A**) Overall qualitative analysis by NGS, representing bacterial genera before and after LGG administration. (**B**) Box-and-whisker plots with data points showing the relative abundances of the genera significantly increased (*p* < 0.05) after LGG administration. Mann–Whitney U Test result of the group comparison is shown in the figure. (**C**) LDA LEfSe barplot displaying the bacterial genera associated with the untreated (T0, in green) subjects; T1 is not visible in the panel since the comparison demonstrated a significant association only at T0 (LDA score > 2.0).

**Figure 4:**
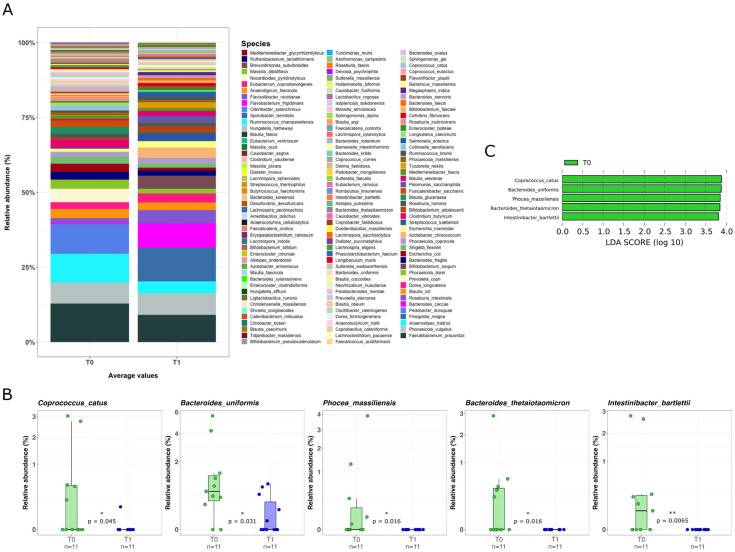
Qualitative analysis at species level of the mucosal microbiota before and after LGG treatment. (**A**) Overall qualitative analysis by NGS, representing bacterial species before and after LGG administration. (**B**) Box-and-whisker plots with data points showing the relative abundances of the species significantly increased (*p* < 0.05) after LGG administration. Mann–Whitney U Test result of the group comparison is shown in the figure. (**C**) LDA LEfSe barplot displaying the bacterial species associated with the untreated (T0, in green) subjects; T1 is not visible in the panel since the comparison demonstrated a significant association only at T0 (LDA score > 2.0).

Given the potential impact of VDR stimulation on gut microbiota composition, we analyzed the mucosal-adherent microbiota by extracting total DNA from colonic biopsies and performing a next-generation sequencing (NGS) analysis. A significant reduction in α-diversity was observed after LGG administration, while β-diversity remained largely unchanged (Figure 2). The reduction in α-diversity was primarily attributed to a decline in commensal bacterial groups. In fact, analyzing genera composition, the commensal bacterial group of *Clostridium*, *Phocea*, *Intestinibacter*, *Lacrimispora*, and *Faecalicatena*, were significantly reduced after LGG administration (*p* < 0.05, Figure 3). Moreover, at species level, we observed a significant reduction in commensal bacteria, including *Coprococcus catus*, *Bacteroides uniformis*, *Phocea massiliensis*, *Bacteroides thetaiotaomicron*, and *Intestinibacter bartlettii* (*p* < 0.05, Figure 4). Besides those findings, due to individual variability and limited samples, despite some differences in the qualitative composition before and after LGG administration, no significant biomarkers could be identified at philum and family level (Supplementary Figure S1).

## 3. Discussion

Vitamin D and probiotics have been independently recognized for their immunomodulatory and gut-homeostasis-promoting effects. Recent preclinical data suggest that, in addition to their separate functions, these two nutraceuticals may exert a synergistic effect on mucosal immune modulation and microbiota regulation. Notably, Wu et al. demonstrated that LGG and *Lactobacillus plantarum* stimulate VDR expression and activity in various cell lines. Furthermore, the protective effect of probiotics against Salmonella-induced colitis was completely abolished in VDR-knockout (KO) mice, suggesting a critical role for VDR activation in probiotic-mediated gut protection [16]. Additionally, probiotics have been shown to enhance VDR expression and activity in preclinical models [17,18,19,20]. However, no previous human studies have confirmed these findings in vivo.

**Figure 5:**
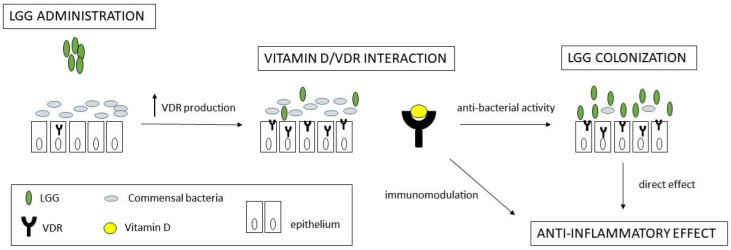
Schematic representation of theoretical working hypothesis for molecular effect of LGG administration in UC patients’ mucosa involving vitamin D/VDR pathway activation. LGG promotes VDR expression, leading to enhanced vitamin D signalling and subsequent modulation of mucosal immune responses. This activation may create a positive feedback loop in which VDR stimulation strengthens the intestinal barrier, downregulates pro-inflammatory pathways, and regulates microbiota composition. In particular, VDR activation has been linked to the production of antimicrobial peptides, which may reduce the concentration of certain commensal bacterial species while facilitating the mucosal colonization of LGG. The enhanced colonization of LGG, in turn, may further stimulate VDR expression and activate downstream anti-inflammatory pathways, contributing to an overall reduction in disease activity.

To our knowledge, this is the first study providing molecular evidence of VDR upregulation following probiotic administration. Based on our findings and on speculation from previously available data, we propose a theoretical working model in which LGG promotes VDR expression, leading to enhanced vitamin D signalling and subsequent modulation of mucosal immune responses. This activation may create a positive feedback loop in which VDR stimulation strengthens the intestinal barrier, downregulates pro-inflammatory pathways, and regulates microbiota composition. In particular, VDR activation has been linked to the production of antimicrobial peptides such as cathelicidin and defensins [21], which may reduce the concentration of certain commensal bacterial species while facilitating the mucosal colonization of LGG. The enhanced colonization of LGG, facilitated by the direct production of anti-bacterial products by LGG and by its remarkable adhesive property [22], in turn, may further stimulate VDR expression and activate downstream anti-inflammatory pathways, contributing to an overall reduction in disease activity (Figure 5).

While many studies suggest that both probiotics and vitamin D contribute to increased microbiota diversity [23,24], our findings indicate a reduction in α-diversity following LGG treatment. This discrepancy may be explained by differences between fecal and mucosal microbiota that present specific microbial signatures [25,26]. Most available data on microbiota dynamics is derived from fecal samples, whereas mucosal-adherent microbiota has been less extensively characterized. Consistently, Uronis et al. demonstrated distinct microbial communities at luminal versus mucosal sites and a probiotic-induced reduction in diversity and richness in fecal microbiota, with no remarkable effect in mucosal microbiota, in a TNBS-induced colitis model [27]. In the present study, we observed a global reduction in the concentration of some commensal bacteria in the colonic mucosa. Considering that a crucial factor for probiotic efficacy is the adhesion and effective colonization of the mucosa, that regulative effect may be a major determinant of the beneficial effect of LGG in UC patients. In fact, in a spontaneous model of Crohn’s disease, inflammation onset was almost completely prevented in SAMP1/YitFc mice who received a very high dose of probiotics, causing an effective mucosal colonization, while such effect was not observed in mice receiving a regular dose of probiotics, with ineffective colonization of the supplemented bacteria [28]. Indeed, the NGS analysis is not the appropriate method to assess the colonization of exogenously assumed bacteria, since the total amount remains very low comparing with other resident species. In order to assess LGG colonization, RT-qPCR with specific primers should be a more appropriate test. We have previously demonstrated ex vivo and in vivo mucosal colonization of LGG in colonic mucosa of UC patients by RT-qPCR, regardless of the inflammatory state, in analogue experiments [12]. In the present study, we chose to prioritize novel investigations, extracting RNA from bioptic samples for gene expression analysis and DNA for the global microbiota evaluation, and therefore we did not confirm the effective colonic mucosal colonization of LGG. Nonetheless, considering our previous findings, we could rationally speculate that we had effective LGG colonization in the present experiment as well, although that was not effectively demonstrated.

Despite these promising findings, the present investigation represents a preliminary proof-of-concept study and has some limitations. First of all, the sample size was small, and further research with larger cohorts is needed to confirm our results. Nonetheless, the increment of VDR expression and production was well demonstrated by two different methodologies (RT-PCR and immunohistochemistry), and with consistent individual difference before vs. after LGG administration. Although no semi-quantitative scoring (i.e., H-score) was performed due to the limited number of samples and the exploratory nature of the study, all the analyzed samples displayed consistent increase in VDR detection after LGG treatment at the immunohistochemical examination. The modification on mucosal microbiota is less striking and needs further specific investigations. Moreover, the effect of LGG should be evaluated in patients with more severe disease activity and over longer treatment durations to assess the full impact of the probiotic bacteria on microbiota composition. Finally, the precise molecular mechanisms underlying probiotic-induced VDR activation warrant further investigation, and the causative relation for the positive clinical effect of LGG and vitamin D/VDR pathway activation needs to be fully proven.

Considering the exploratory nature of the present study, we did not perform a formal a priori power calculation. Nonetheless, in order to better contextualize the strength of our findings, we performed a post hoc power analysis for the primary molecular endpoint and considered exploratory aspects related to the microbiota data. A post hoc power analysis was performed for the paired comparison of VDR mRNA expression before and after LGG administration. Based on the observed mean difference of 2.4 ± 2.2 (*n* = 10), corresponding to a Cohen’s *d* of 1.09, and assuming a two-tailed paired *t*-test with α = 0.05, the achieved statistical power (1−β) was approximately 0.87. This indicates that, for the observed effect size, the study had about an 87% probability of detecting a true difference in VDR expression. Nevertheless, this analysis should be interpreted with caution, as post hoc power is inherently dependent on the observed effect and does not replace prospective sample size estimation. The current work was not designed for hypothesis testing but rather as a proof-of-concept investigation aimed at exploring the feasibility of probiotic-induced VDR modulation in ulcerative colitis. Since we analyzed a subgroup of the LGGinUC trial, that was an uncontrolled trial, the present findings should be better tested and confirmed in a placebo-controlled study. Based on our observed effect on VDR mRNA, for a randomized, parallel-group trial designed to test the same effect, about 25 subjects per arm would be required assuming d = 0.8 (80% power). Due to the multivariate and compositional nature of microbiota data, a formal post hoc power calculation was not feasible. Future studies with larger cohorts will allow formal estimation of effect sizes and power for specific diversity indices and bacterial taxa.

Indeed, a future study investigating potential probiotic/vitamin D interaction should address and consider some variables that were not included in the present work due to its exploratory nature: serum 25 (OH)D levels, sun exposure, diet and medication, genetic polymorphisms (i.e., for CYP27B1 and CYP24A1), and assessment and measurement of mucosal anti-bacterial peptide (i.e., cathelicidins, defensins).

In conclusion, this preliminary proof-of-concept study provides the first molecular evidence in humans that LGG administration in UC patients stimulates the vitamin D/VDR pathway, potentially contributing to its beneficial effects. Understanding the interplay between probiotics and vitamin D may open new avenues for IBD management and warrant further research in this rapidly evolving field.

## 4. Materials and Methods

### 4.1. Patients and Bioptic Samples

Study protocol of the LGGinUC trial has been previously described [8]. In brief, UC patients with mild-to-moderate clinical activity taking only oral mesalamine, after two weeks of stopping mesalamine, were randomized to assume a regular (1.2 × 10^10^ CFU/day, 2 capsules a day) or a double (2.4 × 10^10^ CFU/day, 4 capsules a day) dose of *Lactobacillus rhamnosus* GG (ATCC 53103) for one month. Rectosigmoidoscopy was performed before (t0) and after (t1) treatment, and bioptic samples from the rectum were collected and, after being rinsed twice with sterile physiological solution, put in RNA and later stored at −20 °C until processing.

The study was approved by the local ethic committee (protocol number: 0127710) and was registered to the ClincalTrials.gov website (Identifier: NCT04102852).

For this study, we included 13 patients in the present analysis in whom paired rectal biopsies (pre- and post-treatment) of sufficient quality and quantity were available for both RNA and DNA extraction. No selection based on treatment response or clinical characteristics was performed.

### 4.2. DNA Extraction from Human Biopsies

Total DNA for metagenomic analysis was extracted from colonic biopsies of IBD patients using TRIzol reagent (15596026, Invitrogen, Life Technologies, Milan, Italy) according to the manufacturer’s instructions. Briefly, colonic tissue was mechanically lysed in 500 µL of TRIzol reagent using a Pulse 150 UltraSonic Homogenizer (Benchmark Scientific, Sayreville, NJ, USA). Lysate was centrifuged for 5 min at 12.000× *g* at 4 °C, then clear supernatant was transferred to a new tube and incubated for 5 min. After adding chloroform, samples were centrifuged for 15 min at 12.000× *g* at 4 °C in order to separate the mixture in an upper aqueous phase containing the RNA and an intermediate and lower organic phase for DNA purification. DNA was precipitated in 100% ethanol by 5 min centrifugation at 12.000× *g* at 4 °C; the pellet was resuspended and incubated in 0.1 M sodium citrate in 10% ethanol at pH 8.5 for 30 min and then centrifuged for 5 min at 2000× *g* at 4 °C. The pellet was resuspended and incubated in 75% ethanol for 20 min and centrifuged for 5 min at 2000× *g* at 4 °C before discarding supernatant and air-drying the final DNA pellet, which was resuspended in appropriate volume of 8 mM NaOH and sent to the next sequencing analysis.

### 4.3. Gene Expression Analysis

Expression levels of vitamin D receptor (VDR), before and after LGG therapy, were evaluated by means of quantitative reverse transcription polymerase chain reaction (RT-qPCR). Isolation of mRNAs was performed using TRIzol reagent (15596026, Invitrogen, Life Technologies, Milan, Italy) according to the manufacturer’s instructions. Extracted RNAs were quantified spectrophotometrically (NanoQuant plate on Tecan Infinite M200-Pro, Mannedorf, Switzerland), and RNA purity was evaluated by calculation of A260/A280 absorbance ratio. Among 13 samples, 10 displayed a ratio between 1.8 and 2.1 and were therefore further analyzed. An amount of 1 µg of total RNA was retrotranscribed using the High-capacity cDNA reverse transcription kit (4374967, Thermo Fisher Scientific, Waltham, MA, USA) according to the manufacturer’s instructions. qPCR was performed on 20 ng of cDNA in a total reaction volume of 10 µL on an ABI 7900HT fast real-time PCR System (Thermo Fisher Scientific, Waltham, MA, USA) using TaqMan universal Master Mix II (4440040, Life Technologies, Milan, Italy) and TaqMan Gene Expression Assay primers (Life Technologies, Milan, Italy) for VDR (Hs01045843_m1). An analysis of expression levels of a panel of reference genes was performed through geNorm v.3.5 [29] on 18s rRNA (Hs99999901_s1), ActB (Hs01060665_g1), TBP (Hs00427620_m1), and PPIA (Hs99999904_m1), with the latter two genes selected as endogenous controls.

### 4.4. Immunohistochemistry on Paraffin-Embedded Tissue Sections

FFPE tissue samples were dewaxed in Xylene (28973.328, Avantor Inc., Radnor, PA, USA) and treated with Citrate buffer at pH.6.0 and 98 °C for 30 min. for the antigen retrieval. Immunostaining detection of VDR was performed by using the STAT-Q™ STAINING SYSTEM (# NB314KLD, Innovex Bioscience, Richmond, CA, USA) using the anti-VDR mouse monoclonal Ab (D-6, sc-13133, Santa Cruz Biotechnology, Dallas, TX, USA) diluted 1:100. Nuclear counterstaining was performed by using Mayer’s Hematoxylin (05-M06002, Bio-Optica, Milan, Italy). Slides were examined using the Olympus BX51TF microscope (Olympus, Tokyo, Japan). Images were recorded using a MicroPublisher 6 TM digital camera (Olympus, Tokyo, Japan).

### 4.5. Determination of Bacterial Profiles by Amplicon Sequencing

Negative controls were PCR-grade water (no sample). DNA was quantified using the Qubit™ 4 Fluorometer (Fisher Scientific Italia, Segrate, Italy) following the Illumina amplicon sequencing Sample Preparation Guide (https://emea.support.illumina.com/content/dam/illumina-support/documents/documentation/chemistry_documentation/16s/16s-metagenomic-library-prep-guide-15044223-b.pdf), accessed on the 8 February 2024. V3 to V4 region of the 16S rRNA gene was amplified for bacterial classification using the primer set S-D-Bact-0341-b-S-17/S-D-Bact-0785-a-A-21 [30]. Indexed libraries were prepared on the Hamilton Vantage (Hamilton) automated platform following the Illumina protocol. Limited-cycle PCR was performed using Nextera technology (Illumina, San Diego, CA, USA) and clean up was carried out using the VAHTS DNA Clean Beads (Vazyme, Red Maple Hi-tech Industry Park, Nanjing, China). Libraries were pooled at equimolar concentrations (4 nM), denatured, and diluted to 5 pM before loading onto the MiSeq (Illumina, San Diego, CA, USA). Sequencing on the MiSeq platform was performed by using a 2 × 300 bp paired end protocol.

### 4.6. Data Processing and Analysis

Raw sequences were processed using a pipeline that combines PANDAseq [30] and QIIME2 [31]. The high-quality reads were grouped into Amplicon Sequence Variants (ASV) using the DADA2 [31] (Divisive Amplicon Denoising Algorithm 2) plugin. For the bacterial fraction, the taxonomy was assigned using a custom database containing high-quality 16S sequences retrieved from NCBI, Silva and Greengens (v. May 2012).

The data were imported into R (version 4.2.2) on Rstudio (version 2022.07.2 Build 576), where downstream analysis was performed using R packages phyloseq, rbiom, ggplot2, tidyverse, tidyr, ape, ggpubr, and dplyr. Environmental microbial contaminants were excluded from the present analysis by filtering out ASVs that were specifically present in the negative controls (no sample) using the decontam R package at 5% stringency. Data were not rarefied to similar sequencing depth to avoid any potential loss of information because the number of observed species had reached an optimal value (plateau) for every sample in the study. The minimum sample quality-filtered sequence depth was 5051 reads. The differences in alpha diversity were evaluated using ANOVA and Tukey’s HSD (honestly significant difference) tests for normally distributed data or Wilcoxon-Mann–Whitney with Holm–Bonferroni correction method for non-normally distributed data. Beta diversity was measured by calculating the weighted or unweighted UniFrac distance metric. Principal coordinate analysis (PCoA) was applied on the distance matrices to generate bi-dimensional plots in R. Dispersion of the PCoA clusters was compared using the betadisper function in the R vegan package. The permutational analysis of variance (PERMANOVA) test, calculated using the function adonis2 in the vegan package, was performed to determine whether there was a significant separation between different sample groups. Linear discriminant analysis (LDA) effect size (LEfSe) algorithm [31], a tool which is hosted on the Galaxy web application at http://mbac.gmu.edu:8080/ (accessed on 25 November 2024), was used to discover bacterial taxa associated with each condition. The differences in abundance were regarded as significant when the logarithmic LDA score was higher than two.

### 4.7. Statistical Analysis

The Permutational multivariate analysis of variance (PERMANOVA, 999 permutations) was used to test the difference among groups of microbial beta diversity. Categorical variables are presented as counts and percentages, continuous variables as median, minimum, and maximum values. For group comparisons, the Shapiro–Wilk’s test or the Kolmogorov–Smirnov Test of Normality were used to test data for normality assumptions. Fisher’s exact test was used to analyze categorical variables, the Wilcoxon signed rank and Mann–Whitney U test were used on non-normally distributed continuous data, and the T-Statistical tests were adjusted for multiple comparison where indicated, and was performed on normally distributed continuous data, with a *p* value < 0.05 considered statistically significant. The experimental data were analyzed using Prism ver. 9.3.0 (GraphPad Software, San Diego, CA, USA) and reported as mean ± SD, if not otherwise specified, of the three independent experiments.

## Figures and Tables

**Table 1:** Characteristics of the total UC patients included in the original study and of the subgroup included in the present study. No significant difference emerged between the two groups. * = per protocol analysis; ** = in the subgroup of patients who had endoscopic evaluation pre- and post-LGG treatment.

Characteristics	Original Study (*n* = 76)	Present Study (*n* = 13)
Age (yrs)	57 ± 15	53 ± 16
Gender (M/F)	37/39	7/6
Disease duration (yrs)	9.6 ± 7.1	7.2 ± 5.4
Extension:		
Proctitis Proctosigmoiditis Pancolitis	12 (16%) 41 (54%) 23 (30%)	4 (31%) 7 (54%) 2 (15%)
Partial Mayo:		
2 3 4	62 (82%) 10 (13%) 4 (5%)	11 (85%) 2 (15%) 0
LGG dose:		
Regular Double	38 (51%) 37 (49%)	7 (54%) 6 (46%)
Clinical outcome *:		
Response Stable Flare	32/52 (58%) 21/52 (38%) 2/52 (2%)	6 (46%) 7 (54%) 0
Endoscopic outcome **:		
Response Stable Worse	7/27 (26%) 19/27 (70%) 1/27 (4%)	3 (23%) 9 (69%) 1 (8%)

## Data Availability

The original contributions presented in this study are included in the article/Supplementary Material. Further inquiries can be directed to the corresponding author.

## Supplementary Materials

The following supporting information can be downloaded at: https://www.mdpi.com/article/10.3390/ph18111651/s1, Figure S1: Qualitative analysis by NGS before and after LGG administration. Due to limited samples and internal variability we did not observe significant difference at philum (A) and family (B) level.

## References

[1] Iliev I.D., Ananthakrishnan A.N., Guo C.-J. (2025). Microbiota in inflammatory bowel disease: Mechanisms of disease and therapeutic opportunities. Nat. Rev. Microbiol..

[2] Le Berre C., Honap S., Peyrin-Biroulet L. (2023). Ulcerative colitis. Lancet.

[3] Raine T., Bonovas S., Burisch J., Kucharzik T., Adamina M., Annese V., Bachmann O., Bettenworth D., Chaparro M., Czuber-Dochan W. (2022). ECCO Guidelines on Therapeutics in Ulcerative Colitis: Medical Treatment. J. Crohn’s Colitis.

[4] Kaur L., Gordon M., Baines P.A., Iheozor-Ejiofor Z., Sinopoulou V., Akobeng A.K. (2020). Probiotics for induction of remission in ulcerative colitis. Cochrane Database Syst. Rev..

[5] Iheozor-Ejiofor Z., Kaur L., Gordon M., Baines P.A., Sinopoulou V., Akobeng A.K. (2020). Probiotics for maintenance of remission in ulcerative colitis. Cochrane Database Syst. Rev..

[6] Capurso L. (2019). Thirty Years of *Lactobacillus rhamnosus* GG: A Review. J. Clin. Gastroenterol..

[7] Zocco M.A., dal Verme L.Z., Cremonini F., Piscaglia A.C., Nista E.C., Candelli M., Novi M., Rigante D., Cazzato I.A., Ojetti V. (2006). Efficacy of Lactobacillus GG in maintaining remission of ulcerative colitis. Aliment. Pharmacol. Ther..

[8] Pagnini C., Di Paolo M.C., Urgesi R., Pallotta L., Fanello G., Graziani M.G., Fave G.D. (2023). Safety and Potential Role of *Lactobacillus rhamnosus* GG Administration as Monotherapy in Ulcerative Colitis Patients with Mild-Moderate Clinical Activity. Microorganisms.

[9] Kankainen M., Paulin L., Tynkkynen S., von Ossowski I., Reunanen J., Partanen P., Satokari R., Vesterlund S., Hendrickx A.P.A., Lebeer S. (2009). Comparative genomic analysis of *Lactobacillus rhamnosus* GG reveals pili containing a human- mucus binding protein. Proc. Natl. Acad. Sci. USA.

[10] Donato K.A., Gareau M.G., Wang Y.J.J., Sherman P.M. (2010). *Lactobacillus rhamnosus* GG attenuates interferon-gamma and tumour necrosis factor-alpha-induced barrier dysfunction and pro-inflammatory signalling. Microbiology.

[11] Yan F., Cao H., Cover T.L., Washington M.K., Shi Y., Liu L., Chaturvedi R., Peek R.M., Wilson K.T., Polk D.B. (2011). Colon-specific delivery of a probiotic-derived soluble protein ameliorates intestinal inflammation in mice through an EGFR-dependent mechanism. J. Clin. Investig..

[12] Pagnini C., Corleto V.D., Martorelli M., Lanini C., D’ambra G., Di Giulio E., Fave G.D. (2018). Mucosal adhesion and anti-inflammatory effects of *Lactobacillus rhamnosus* GG in the human colonic mucosa: A proof-of-concept study. World J. Gastroenterol..

[13] Wang J., Mei L., Hao Y., Xu Y., Yang Q., Dai Z., Yang Y., Wu Z., Ji Y. (2024). Contemporary Perspectives on the Role of Vitamin D in Enhancing Gut Health and Its Implications for Preventing and Managing Intestinal Diseases. Nutrients.

[14] Del Pinto R., Ferri C., Cominelli F. (2017). Vitamin D Axis in Inflammatory Bowel Diseases: Role, Current Uses and Future Perspectives. Int. J. Mol. Sci..

[15] Pagnini C., Di Paolo M.C., Graziani M.G., Fave G.D. (2021). Probiotics and Vitamin D/Vitamin D Receptor Pathway Interaction: Potential Therapeutic Implications in Inflammatory Bowel Disease. Front. Pharmacol..

[16] Wu S., Yoon S., Zhang Y.-G., Lu R., Xia Y., Wan J., Petrof E.O., Claud E.C., Chen D., Sun J. (2015). Vitamin D receptor pathway is required for probiotic protection in colitis. Am. J. Physiol. Gastrointest. Liver Physiol..

[17] Appleyard C.B., Cruz M.L., Isidro A.A., Arthur J.C., Jobin C., De Simone C. (2011). Pretreatment with the probiotic VSL#3 delays transition from inflammation to dysplasia in a rat model of colitis-associated cancer. Am. J. Physiol. Gastrointest. Liver Physiol..

[18] Mencarelli A., Cipriani S., Renga B., Bruno A., D'Amore C., Distrutti E., Fiorucci S. (2012). VSL#3 resets insulin signaling and protects against NASH and atherosclerosis in a model of genetic dyslipidemia and intestinal inflammation. PLoS ONE.

[19] Raveschot C., Coutte F., Frémont M., Vaeremans M., Dugersuren J., Demberel S., Drider D., Dhulster P., Flahaut C., Cudennec B. (2020). Probiotic Lactobacillus strains from Mongolia improve calcium transport and uptake by intestinal cells in vitro. Food Res. Int..

[20] Wang H., Tian G., Pei Z., Yu X., Wang Y., Xu F., Zhao J., Lu S., Lu W. (2025). Bifidobacterium longum increases serum vitamin D metabolite levels and modulates intestinal flora to alleviate osteoporosis in mice. mSphere.

[21] White J.H. (2022). Emerging Roles of Vitamin D-Induced Antimicrobial Peptides in Antiviral Innate Immunity. Nutrients.

[22] Leser T., Baker A. (2024). Molecular Mechanisms of *Lacticaseibacillus rhamnosus*, LGG^®^ Probiotic Function. Microorganisms.

[23] Grazul H., Kanda L.L., Gondek D. (2016). Impact of probiotic supplements on microbiome diversity following antibiotic treatment of mice. Gut Microbes.

[24] Singh P., Rawat A., Alwakeel M., Sharif E., Al Khodor S. (2020). The potential role of vitamin D supplementation as a gut microbiota modifier in healthy individuals. Sci. Rep..

[25] Zmora N., Zilberman-Schapira G., Suez J., Mor U., Dori-Bachash M., Bashiardes S., Kotler E., Zur M., Regev-Lehavi D., Brik R.B.-Z. (2018). Personalized Gut Mucosal Colonization Resistance to Empiric Probiotics Is Associated with Unique Host and Microbiome Features. Cell.

[26] Rangel I., Sundin J., Fuentes S., Repsilber D., de Vos W.M., Brummer R.J. (2015). The relationship between faecal-associated and mucosal-associated microbiota in irritable bowel syndrome patients and healthy subjects. Aliment. Pharmacol. Ther..

[27] Uronis J.M., Arthur J.C., Keku T., Fodor A., Carroll I.M., Cruz M.L., Appleyard C.B., Jobin C. (2011). Gut microbial diversity is reduced by the probiotic VSL#3 and correlates with decreased TNBS-induced colitis. Inflamm. Bowel Dis..

[28] Pagnini C., Saeed R., Bamias G., Arseneau K.O., Pizarro T.T., Cominelli F. (2010). Probiotics promote gut health through stimulation of epithelial innate immunity. Proc. Natl. Acad. Sci. USA.

[29] Vandesompele J., De Preter K., Pattyn F., Poppe B., Van Roy N., De Paepe A., Speleman F. (2002). Accurate normalization of real-time quantitative RT-PCR data by geometric averaging of multiple internal control genes. Genome Biol..

[30] Masella A.P., Bartram A.K., Truszkowski J.M., Brown D.G., Neufeld J.D. (2012). PANDAseq: Paired-end assembler for illumina sequences. BMC Bioinform..

[31] Hall M., Beiko R.G. (2018). 16S rRNA Gene Analysis with QIIME2. Methods Mol. Biol..

