# Supplementary material for: Lactobacillus rhamnosus GG Administration Is Associated with Stimulation of Vitamin D/VDR Pathway and Mucosal Microbiota Modulation in Ulcerative Colitis Patients: A Pilot Study
Source: Pharmaceuticals (Basel). 2025 Nov 1;18(11):1651. doi: 10.3390/ph18111651 (PMC12655305; doi:10.3390/ph18111651)
Supplement: Supplementary file 1 [file pharmaceuticals-18-01651-s001.zip › pharmaceuticals-3917686-supplementary.pdf]

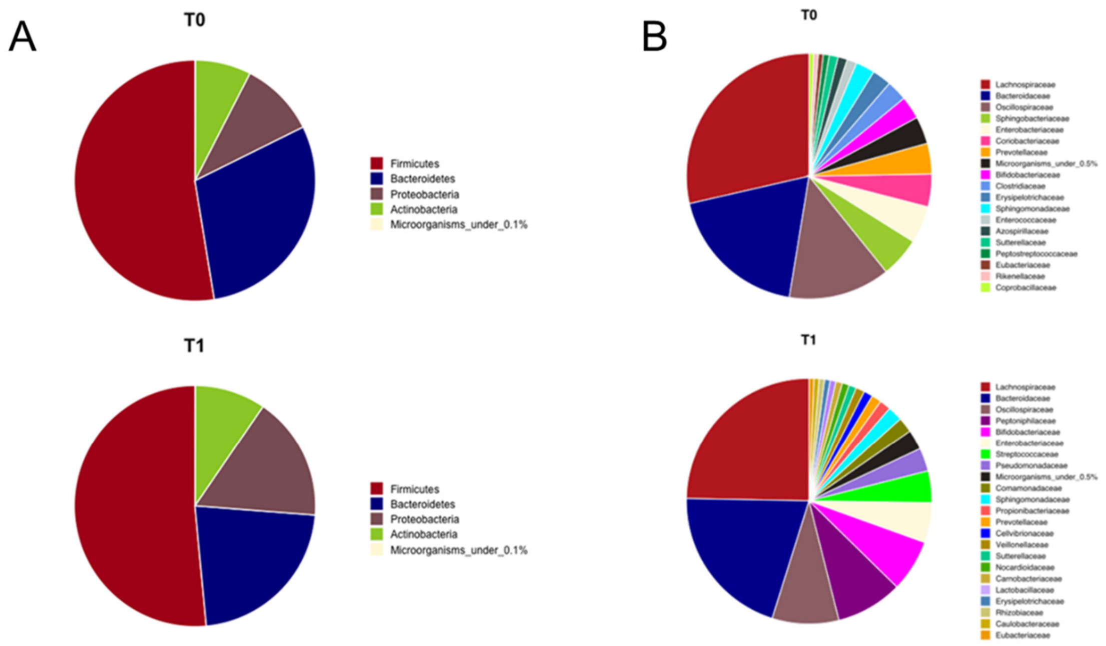

Supplementary Figure S1 – Qualitative analysis by NGS before and after LGG administration. Due to limited samples and internal variability we did not observe significant difference at philum (A) and family (B) level.
